# SARC-T a new physical test for sarcopenia assessment with development, validation and physiological evaluation

**DOI:** 10.3389/fragi.2026.1649622

**Published:** 2026-03-02

**Authors:** Blanca Pedauyé-Rueda, José Luis Maté-Muñoz, Juan Hernández-Lougedo, Iñigo Aparicio-García, Sara Cerrolaza-Tudanca, Manuel Rozalén-Bustín, Inmaculada Rodríguez-Moreno, Pablo García-Fernández

**Affiliations:** 1 Physiotherapy and Health Research Group (FYSA), Department of Physiotherapy, HM Faculty of Health Sciences, Camilo José Cela University, Madrid, Spain; 2 HM Hospitals Health Research Institute, Madrid, Spain; 3 Faculty of Nursing, Physiotherapy and Podiatry, Complutense University of Madrid, Madrid, Spain; 4 Department of Physical Activity and Sports Science, Alfonso X El Sabio University, Madrid, Spain; 5 Emera Group Elderly Care Home, Madrid, Spain

**Keywords:** aging, assessment, chair test, physical test, sarcopenia

## Abstract

**Background:**

Sarcopenia is a disease characterized by the progressive loss of muscle mass and strength associated with aging. There are marked differences in sarcopenia prevalence depending on the diagnostic algorithm used. It has been demonstrated that muscle power is the most relevant predictor for determining functional limitations in older adults. The objectives of this study were to evaluate the performance of the Sarcopenia Optoelectronic Chair-Rise Velocity Test (SARC-T) as complementary alternative to tests that determinate probable sarcopenia and/or assess its severity, as well as to assessment analyze its correlation with other validated tests.

**Methods:**

A cross-sectional analysis was conducted in a population residing in elderly care centers. All physical tests included in the second version of the diagnostic algorithm developed by the European Working Group on Sarcopenia in Older People 2 (EWGSOP2) in 2019 were performed. In addition, the SARC-T was administered to measure the speed at which participants rose from a chair. Physiological variables, including heart rate (HR), systolic blood pressure (SBP), and oxygen saturation (SpO_2_), were also monitored.

**Results:**

The sarcopenia group showed significantly lower physical performance than the non-sarcopenia group in all tests. At a physiological level, no significant differences were found between groups in the 5-STST, Handgrip, and TUG. Additionally, the SARC-T showed a strong correlation with Handgrip (r = 0.800), 5-STST (r = −0.719) and TUG (r = −0.523), and a moderate correlation with Gait Speed (r = −0.438) in sarcopenia group.

**Conclusion:**

The SARC-T could be a safe, accurate, and low-impact complementary tool for assessing the probability and severity of sarcopenia.

## 1 Introduction

Sarcopenia is a condition characterized by the progressive loss of muscle mass and strength associated with aging (Rosenberg, 1997). However, the decline in muscle strength occurs at a faster rate than the reduction in muscle mass in older individuals (Goodpaster et al., 2006). In 2016, sarcopenia was officially recognized as a disease and included in the International Classification of Diseases (ICD) under the ICD-10-CM code M62.84 (Anker et al., 2016).

This condition is associated with multiple adverse health outcomes, including disability and functional decline (Janssen et al., 2002), an increased risk of falls (Scott et al., 2014), and higher mortality rates (Westbury et al., 2023). These clinical consequences lead to a significant increase in healthcare costs. In 2014, individuals diagnosed with sarcopenia generated an additional annual healthcare expenditure of approximately $2,315.7 per person compared to those without the condition (Goates et al., 2019).

The most commonly used diagnostic algorithms for sarcopenia have been developed by the European Working Group on Sarcopenia in Older People (EWGSOP) (Cruz-Jentoft et al., 2019), the Asian Working Group for Sarcopenia (AWGS) (Chen et al., 2020), and the Foundation for the National Institutes of Health (FNIH) (Studenski et al., 2014). Additionally, other diagnostic approaches have been proposed by the International Working Group on Sarcopenia (IWGS) (Fielding et al., 2011) and the Sarcopenia Definitions and Outcomes Consortium (SDOC) (Bhasin et al., 2020). Each of these algorithms incorporates different functional tests, leading to variations in diagnostic procedures, as well as the need for specific equipment and time for implementation.

One of the main reasons for discrepancies in sarcopenia prevalence is the heterogeneity in diagnostic tests, established cutoff points, and the equipment used to depend on the applied algorithm (Pedauyé-Rueda et al., 2024). In scientific literature, sarcopenia prevalence varies widely, ranging from 9.9% to 40.4%, with an increasing trend as age progresses (Chang et al., 2020). These differences can be attributed to the use of different diagnostic algorithms and variability in tests assessing muscle strength, such as the 5-Sit-To-Stand Test (5-STST) and handgrip dynamometry (Celis-Morales et al., 2018; Cofré-Bolados et al., 2021). Two studies using the EWGSOP algorithm reported different prevalence rates depending on the test used to assess muscle strength: in one study, prevalence was 3.1% when applying the 5-STST (Johansson et al., 2020), whereas another study found a 3% higher prevalence when using handgrip dynamometry (Yee et al., 2021).

Muscle power has been identified as the most relevant predictor of functional limitations in older adults (Martinikorena et al., 2016). Various studies have employed equations incorporating variables such as leg length, chair height, and execution time to estimate muscle power. However, these methods have not been validated against reference instruments (Takai et al., 2009; Yanagawa et al., 2015). Additionally, equations have been developed to estimate power as a means of providing cost-effective and easily applicable tools for assessing sarcopenia and functional capacity in geriatric populations. Nevertheless, these equations have not yet replaced previously validated methods (Alcazar et al., 2018b).

Other methodologies for assessing muscle strength include the One-Repetition Maximum (1RM) test, the Countermovement Jump (CMJ), and the maximum number of repetitions with a fixed load test. However, their application in older adults is limited due to their high physical demands, induced fatigue, and potential physiological alterations (Rodrigues et al., 2024). Consequently, these methods have primarily been restricted to sports performance assessment (Alcazar et al., 2018a).

Current sarcopenia case-finding and diagnostic pathways, including the European Working Group on Sarcopenia in Older People 2 (EWGSOP2) algorithm, rely on functional tests such as handgrip strength, the 5-times sit-to-stand test, gait speed and the Timed Up and Go. However, in real-world geriatric settings these assessments may be limited by pain, balance impairment, mobility restrictions and fatigue, and they can be impractical when space, time or equipment are constrained. Moreover, several widely used outcomes depend on manual timing or indirect calculations, which may reduce precision and comparability across settings. Although muscle power and movement velocity are closely related to functional limitation in older adults, they are rarely quantified directly in routine sarcopenia assessment. This highlights an unmet need for a brief, low-burden, objective and instrumented test that can complement existing algorithms and provide an operational alternative when standard tests are not feasible or are contraindicated. To address this gap, we developed the Sarcopenia Optoelectronic Chair-Rise Velocity Test (SARC-T), which quantifies mean propulsive velocity during a chair-rise task using a validated optoelectronic encoder.

To support implementation in clinical and research settings, the measurement properties of SARC-T should be evaluated using an accepted framework for outcome measurement instruments. The COSMIN initiative provides consensus-based terminology and standards to assess key measurement properties (e.g., validity) across outcome measurement instruments, including performance-based tests. Accordingly, and given that SARC-T is not a patient-reported outcome measure, we focused on validity-related measurement properties that are applicable to performance-based instruments (Mokkink et al., 2010).

In this context, the primary aim of this cross-sectional study was to evaluate construct validity (hypotheses testing) of SARC-T in older adults by examining *a priori* expected correlations between SARC-T mean propulsive velocity and EWGSOP2-recommended functional tests used to estimate probable sarcopenia and severity. A secondary aim was to evaluate known-groups validity by testing whether SARC-T discriminates between older adults classified with sarcopenia and age- and sex-matched non-sarcopenic controls. As an additional objective related to feasibility and safety, we characterised the physiological load of the procedure by recording heart rate, blood pressure, and oxygen saturation before and after testing.

## 2 Materials and methods

### 2.1 Study design and participants

A cross-sectional analysis was conducted on older adults residing in nursing homes in the Community of Madrid between February and June 2023. This study was approved by the Ethics Committee of the Hospital Clínico San Carlos (Spain) under the code: 23/010-E_TFM. All participants provided written informed consent, and the study followed the Strengthening the Reporting of Observational Studies in Epidemiology (STROBE) guidelines for conducting observational studies in epidemiology (von Elm et al., 2008).

The inclusion criteria were as follows: individuals aged 65 years or older, capable of maintaining a standing position, with an adequate understanding of Spanish, and diagnosed with sarcopenia using the EWGSOP2 algorithm. This algorithm includes the completion of the Strength, Assistance with walking, Rise from a chair, Climb stairs, and Falls (SARC-F) questionnaire and the administration of the following tests: Handgrip (HG)and 5-Sit-to-Stand Test (5-STST) to determinate probable sarcopenia, assessment of musculoskeletal mass using bioimpedance or dual-energy X-ray absorptiometry, to confirm the diagnosis of sarcopenia, and Gait Speed (GS) and Timed Up and Go (TUG) to determinate the severity of the condition.

The exclusion criteria included refusal to participate in the study, failure to sign informed consent, visual and/or auditory impairment that prevented understanding of the tests, use of psychoactive substances affecting test performance, and any orthopedic, neurological, and/or cardiorespiratory condition that hindered the participant’s ability to complete the assessments included in the study.

The control group, identified as non-sarcopenic using the EWGSOP2 algorithm, was recruited from the same nursing homes as the intervention group participants. They were matched by sex and age to the intervention group members.

### 2.2 Measurements and physical tests

**FIGURE 1:**
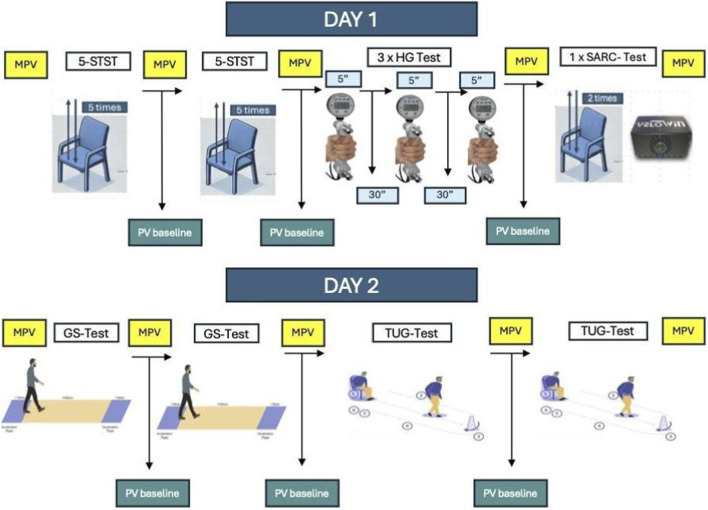
Study design. MPV, Measurement physiological variables; PV, physiological variables; 5-STST, 5-sit-to-stand-test; HG, Handgrip; GS, Gait Speed; TUG, Time Up Go.

Before starting the study, data on age and anthropometric values of the participants were collected. The order of the tests was performed according to a randomization carried out previously. The physiological variables assessed were heart rate (HR), blood pressure (BP) and oxygen saturation (SpO_2_) before, during and after each test, allowing adequate rest periods between tests, allowing oxygen saturation and heart rate to return to baseline values with at least 30 min between tests (Figure 1).

The tests were interrupted in the following situations:

- The participant was unable to complete the test.
- Symptoms such as dizziness, dyspnea or headache appeared.
- 90% of the maximum theoretical heart rate (HRmax) reached HRmax = 210 - (0.65 × age).
- Oxygen desaturation occurred, defined as SpO2 less than 90% or a 4% decrease from baseline.
- The participant voluntarily decided to stop the test.

#### 2.2.1 SARC-F questionnaire

After signing the informed consent form, all participants completed the SARC-F questionnaire. This questionnaire is composed of five questions with a score of 0–2 points each, where 0 = no difficulty, 1 = some difficulty and 2: great difficulty or disability. The questions provide information on strength status, need for aids for walking, getting up from a chair, climbing stairs and prevalence of falls. It was originally proposed with the aim of facilitating early case finding based on self-reported functional limitations related to muscle strength and physical performance. The maximum score is 10 points, a score that represents poorer physical fitness (Malmstrom et al., 2016). Thanks to its ease of administration, low cost, and minimal time commitment, the SARC-F has been widely adopted and recommended by international working groups. The EWGSOP2 uses this questionnaire for case finding and determines that it is positive when a score of 4 or more points is obtained (Cruz-Jentoft et al., 2019).

#### 2.2.2 Anthropometric and body composition values

A stadiometer and a scale (Seca 711, Seca, Hamburg, Germany) were used to record the height and weight of the participants. Skeletal muscle mass was assessed with the XpertZM3 electrical impedance apparatus (Aminogram, La Ciotat - France). The measurement was quadrupole, five multi-frequencies at 200 Hz and with an accuracy of ±1%. The body composition data were transmitted via Bluetooth to the Biody Manager application. The measurement was performed in the retromalleolar area of the right foot, prior to the measurement the area was moistened and then the bioimpedance device was placed so that the electrodes were positioned at an angle of 45° with respect to the calcaneus.

#### 2.2.3 5-sit-to-stand-test

A standard height chair (46 cm) without armrests placed against a wall was used for the test, and participants were instructed that they could not use their hands or arms to push the chair seat or their body. Participants were asked to stand up on 5 occasions as quickly as possible (Cesari et al., 2009). The evaluator timed the time, which started to be measured when the verbal signal ‘go’ was given and ends when the participant sits down for the fifth time. According to the EWGSOP2, when the subject performs the test in a time longer than 15 s, the subject has probable sarcopenia (Cruz-Jentoft et al., 2019).

#### 2.2.4 Handgrip

The test was performed according to the recommendations of the European Society for Clinical and Economic Aspects of Osteoporosis, Osteoarthritis and Musculoskeletal Diseases (ESCEO) (Beaudart et al., 2019). To perform the test, participants were asked to sit in a chair, with their arms resting on the armrest, take the Saehan DHD-1 digital dynamometer (Saehan Corporation, Masan Free Trade Zone - South Korea), and place the elbow in 90° flexion, the wrist in 0°–30° dorsiflexion and 0°–15° ulnar deviation. Participants performed three 5-s dominant hand repetitions with a 30-s rest in between (Beseler et al., 2014). The value used to evaluate the test was the mean value between the three repetitions. The cut-off points for probable sarcopenia according to the EWGSOP2 are <27 kg for men and <16 kg for women (Cruz-Jentoft et al., 2019).

#### 2.2.5 Gait speed

The recommendations of the European Society for Clinical and Economic Aspects of Osteoporosis, Osteoarthritis and Musculoskeletal Diseases (ESCEO) were followed for this test (Beaudart et al., 2019). The start and end points of the test were marked in a corridor, 4 m apart. The subject was instructed to walk as fast as possible from one point to the other and the time was timed from when the first foot was lifted to crossing the end mark. They were left 1 m before and after the start of timing so that they had an acceleration and deceleration zone to avoid braking. The participants performed the test twice, with the necessary rest for the physiological parameters to return to baseline, the value used to be the average of the two repetitions to evaluate this test. According to the EWGSOP2 there is a low walking speed of 0.8 m/s or less (Cruz-Jentoft et al., 2019).

#### 2.2.6 Time Up Go

The test consisted of measuring the time it took participants to get up from a chair with a height of 46 cm, walk 3 m, turn around a cone and sit down again. The participant started with their back against the chair, arms resting on the arms of the chair and the walker within reach. The participant was instructed to stand up and walk to a line on the floor 3 m away, turn around, return to the chair and sit down again (Podsiadlo and Richardson, 1991). Participants performed the test twice, with a rest period necessary for the values of the different physiological parameters to return to baseline. The value used was the mean of the two repetitions. This test was performed to assess physical performance, gait and dynamic balance (Beaudart et al., 2019). The EWGSOP2 indicates that physical performance is low when subjects perform it in a time equal to or greater than 20 s (Cruz-Jentoft et al., 2019).

#### 2.2.7 SARC-T

To carry out this test, the subject was asked to lift a 46 cm high chair twice, without the help of the arms, performing both repetitions at maximum speed and recording the value of the fastest repetition. The started position for the test was standing, with the knees and hips fully extended and the feet shoulder-width apart. We placed a reflective plate on the subject’s deltoid muscle, as an optoelectrical sensor requires a reflective reference mark. Participants were instructed to perform the eccentric phase at a controlled speed (0.45–0.75 m/s) with the help of real-time casual anacoustic feedback provided by the linear velocity transducer software and, from that point, to perform the concentric phase at the maximum possible speed until the starting position. The verbal cue “as fast as you can” was provided to encourage maximum effort. The subject performed two repetitions, and the execution speed of each was measured using a validated and calibrated optoelectronic encoder (Velowin v.1.7.232, Instrumentos y Tecnología Deportiva; Murcia, Spain). The software (Velowin v.1.7.232) calculated the mean propulsive velocity using algorithms (Peña García-Orea et al., 2021).

### 2.3 Sample size

The sample size was estimated *a priori* using G*Power (version 3.1.9.7; Heinrich Heine University Düsseldorf, Düsseldorf, Germany), following published guidance for correlation studies assuming a null correlation of zero (H0: ρ = 0) (Bujang and Baharum, 2016). The study aimed to examine, in older adults with sarcopenia, the association between a progressive lower-limb strength test and the Five Times Sit-to-Stand Test, Timed Up and Go Test, Gait Speed Test, and Hand Grip Strength Test. A two-tailed correlation test (α = 0.05) with 80% power was planned to detect at least a moderate correlation (ρ = 0.50). Accordingly, the minimum required sample size was n = 33 participants with sarcopenia. To account for potential missing/incomplete data (10%), we planned to include at least n = 36 participants. Finally, 38 participants with sarcopenia were included, thus meeting and exceeding the *a priori* requirement. The calculation was based on a two-tailed test (Guenther, 1977).

### 2.4 Statistical analysis

All analyses were performed using SPSS version 29.0 (IBM SPSS Statistics, Armonk, NY, United States). Normality was assessed using the Shapiro–Wilk test. Data are presented as mean ± standard deviation (SD), 95% confidence interval (CI), and minimum and maximum values. Statistical significance was set at p < 0.05 (two-tailed). Following COSMIN terminology for performance-based outcome measurement instruments, construct validity (hypotheses testing) was evaluated by testing *a priori* hypotheses regarding the expected direction and magnitude of associations between SARC-T mean propulsive velocity and EWGSOP2-recommended functional tests (handgrip strength, 5-times sit-to-stand, gait speed, and Timed Up and Go). Associations were quantified using Pearson’s correlation coefficient for normally distributed variables or Spearman’s rho for non-normally distributed variables, and correlation structures were additionally visualised using correlation network graphs. Known-groups validity was assessed by comparing SARC-T outcomes between older adults classified with sarcopenia and age- and sex-matched non-sarcopenic controls; between-group comparisons were performed using an independent-samples t-test (normal distribution) or Mann–Whitney U test (non-normal distribution), and effect sizes were expressed as Hedges’ g with 95% CI. Physiological responses to SARC-T were recorded immediately before and after testing and summarised descriptively. Between-group comparisons at each time point (baseline and post-test) were performed using an independent-samples t-test or Mann–Whitney U test, as appropriate. Within the sarcopenia group, physiological responses across the different functional tests were compared using repeated-measures analysis of variance (ANOVA) with Bonferroni-adjusted post-hoc tests, as appropriate.

## 3 Results

A total of 38 older adults diagnosed with sarcopenia according to the EWGSOP2 algorithm were recruited and compared with 38 healthy subjects. The descriptive characteristics of the participants are presented in Table 1.

**TABLE 1:** Descriptive statistic of the included patients.

Variable	Control group (n = 38)	Sarcopenia group (n = 38)	*p*
Age (years)	87 (85–88)	87 (85–88)	1.000*
Gender Male (n, %) Female (n, %)	18 (47%) 20 (53%)	18 (47%) 20 (53%)	NA
Weight (Kg)	67.7 ± 14.1	59.0 ± 12.3	0.008†
Height (cm)	157.3 ± 6.9	153.0 ± 7.0	0.009†

CI: confidence interval; *: t Student test was used; †:U-Mann-Whitney was used.

Construct validity was evaluated according to COSMIN recommendations by testing *a priori* hypotheses regarding the expected direction and magnitude of the associations between SARC-T mean propulsive velocity and EWGSOP2-recommended functional tests. As shown in Table 2, higher SARC-T velocity was associated with better functional performance in both groups, showing inverse correlations with time-based tests (5-times sit-to-stand, gait speed test time and Timed Up and Go) and positive correlations with handgrip strength. All four *a priori* hypotheses were confirmed in both the control and sarcopenia groups (Table 2), supporting construct validity of SARC-T. Correlation network graphs are provided as supplementary visualisations in Supplementary Material S1 (Supplementary Figures S1, S2).

**TABLE 2:** Construct validity. Associations between SARC-T mean propulsive velocity and EWGSOP2-recommended functional tests.

Functional test	*A priori* hypothesis (direction and magnitude)	Control group (n = 38)	Sarcopenia group (n = 38)	Hypothesis confirmed
		r (95% CI) p	r (95% CI) p	
5-Times sit-to-stand (time)	Inverse, ≥ moderate (r ≥ 0.40)	−0.476 (95% CI -0.691 to −0.184) p = 0.002	−0.719 (95% CI -0.845 to −0.518) p < 0.001	Yes
Gait speed test (time)	Inverse, ≥ moderate (r ≥ 0.30)	−0.452 (95% CI -0.674 to −0.155) p = 0.004	−0.438 (95% CI -0.665 to −0.138) p = 0.005	Yes
Handgrip strength	Positive, strong (r ≥ 0.50)	0.735 (95% CI 0.543–0.854) p < 0.001	0.800 (95% CI 0.645–0.892) p < 0.001	Yes
Timed up and go (time)	Inverse, ≥ moderate (r ≥ 0.40)	−0.591 (95% CI -0.766 to −0.335) p = 0.002	−0.523 (95% CI -0.722 to −0.244) p = 0.002	Yes

Correlations were computed using Pearson’s r (or Spearman’s rho if non-normal, as applicable). 95% CIs, for correlations were derived using Fisher’s z transformation. Time-based tests are expected to show inverse associations with SARC-T, velocity (higher velocity = better performance).

As shown in Table 3, SARC-T mean propulsive velocity was significantly lower in the sarcopenia group than in age- and sex-matched controls (p < 0.001), with a large between-group difference (Hedges’ g = 0.88, 95% CI 0.41–1.35). Consistent between-group differences were also observed in EWGSOP2-recommended functional tests, with the sarcopenia group showing slower performance in time-based tests (Timed Up and Go, gait speed test time, and 5-times sit-to-stand) and lower handgrip strength. Effect sizes ranged from moderate (TUG and gait speed test time) to very large (5-times sit-to-stand; g = −1.73, 95% CI −2.26 to −1.20), further supporting the discriminative capacity (known-groups validity) of SARC-T.

**TABLE 3:** Known-groups validity: Differences in SARC-T and functional test performance between control and sarcopenia groups.

Test	Control group (n = 38) mean (95% CI)	Sarcopenia group (n = 38) mean (95% CI)	p	Hedges’ g (95% CI) (control − sarcopenia)
SARC-T velocity	0.37 (0.33–0.41)	0.28 (0.26–0.31)	<0.001†	0.88 (0.41–1.35)
Timed up and go test time	14.0 (11.5–16.5)	24.6 (15.4–33.8)	0.031†	−0.51 (−0.97–−0.05)
Gait speed test time	5.2 (4.4–5.9)	8.2 (5.5–10.8)	0.034*	−0.50 (−0.96–−0.04)
5-Times sit-to-stand test time (5-STST)	14.0 (12.3–15.6)	23.6 (21.7–25.6)	<0.001*	−1.73 (−2.26–−1.20)
Handgrip strength (kg)	18.9 (17.1–20.8)	12.9 (11.0–14.7)	<0.001*	1.06 (0.57–1.54)

Values are mean (95% CI). Hedges’ g is reported with 95% CI; negative values for time-based tests indicate poorer performance (longer time) in the sarcopenia group. *t-test was used; † Mann–Whitney U test used.

Physiological variables measured before and immediately after SARC-T showed no evidence of meaningful between-group differences in pre–post change scores (Table 4). Specifically, the changes in oxygen saturation, heart rate, systolic blood pressure and diastolic blood pressure were comparable between the control and sarcopenia groups (all p > 0.05). Physiological responses for the remaining functional tests are provided in Supplementary Material S2 (Supplementary Table S1).

**TABLE 4:** Physiological response to SARC-T.

Variable	Control group baseline	Control group final	Δ control	Sarcopenia group baseline	Sarcopenia group final	Δ sarcopenia	p (Δ between groups)
SpO_2_ (%)	95.5 ± 2.1	95.4 ± 2.5	−0.1	96.1 ± 1.7	96.1 ± 3.8	0.0	0.832
HR (bpm)	74.4 ± 13.6	81.4 ± 13.0	+7.0	71.2 ± 11.4	80.0 ± 9.6	+8.8	0.454
SBP (mmHg)	130.5 ± 17.0	131.5 ± 18.5	+1.0	126.4 ± 16.1	129.1 ± 19.7	+2.7	0.597
DBP (mmHg)	72.8 ± 9.8	73.3 ± 8.8	+0.5	71.6 ± 11.6	74.8 ± 10.0	+3.2	0.243

SpO2: oxygen saturation; HR: heart rate; SBP: systolic blood pressure; DBP: diastolic blood pressure; bpm: beats per minute; mmHg: millimeters of mercury. Values are mean ± SD. Δ indicates post–pre change. Between-group differences in Δ were tested using an independent-samples t-test (or Mann–Whitney U if non-normal).

As shown in Table 5, baseline and post-test values of SpO_2_, heart rate and blood pressure were comparable across SARC-T and the other functional tests (TUG, gait speed, 5-STST and handgrip) within the sarcopenia group. No statistically significant differences were observed across tests for any physiological variable at baseline or immediately after testing (all p > 0.05). Importantly, SARC-T did not elicit a greater or more concerning physiological response than the assessments routinely used in sarcopenia evaluation, with no indication of disproportionate desaturation, tachycardia or blood pressure elevations when compared with the other tests. The physiological response across the different functional tests in the control group is presented in Supplementary Material S3 (Supplementary Table S2).

**TABLE 5:** Physiological response across functional tests in the sarcopenia group.

Variables	SARC-T	TUG-test	GS-test	Sarcopenia group (n = 38)	5-STST	*p-value*
				HG-test		
SpO_2_ baseline (%)	96.1 ± 1.7	95.8 ± 1.3	96.1 ± 1.8	97.2 ± 1.9	96.2 ± 1.3	0.841
SpO_2_ final (%)	96.1 ± 3.8	95.5 ± 1.7	96.1 ± 2.4	96.7 ± 3.9	96.1 ± 1.6	0.343
HR baseline (bpm)	71.2 ± 11.4	67.0 ± 11.7	70.3 ± 11.3	73.2 ± 10.4	71.2 ± 9.4	0.952
HR final (bpm)	80.0 ± 9.6	75.2 ± 10.9	77.7 ± 14.0	81.2 ± 9.2	80.2 ± 9.7	0.216
SBP baseline (mmHg)	126.4 ± 16.1	127.6 ± 17.6	128.2 ± 16.5	128.3 ± 15.1	127.3 ± 10.1	0.949
SBP final (mmHg)	129.1 ± 19.7	129.2 ± 20.1	125.7 ± 20.6	130.1 ± 18.7	128.1 ± 16.7	0.176
DBP baseline (mmHg)	71.6 ± 11.6	73.7 ± 9.2	77.7 ± 23.1	70.9 ± 10.5	73.9 ± 8.6	0.665
DBP final (mmHg)	74.8 ± 10.0	73.1 ± 9.2	70.4 ± 17.7	75.8 ± 9.0	73.5 ± 8.1	0.541

SpO2, oxygen saturation; HR, heart rate; SBP, systolic blood pressure; DBP, diastolic blood pressure; bpm, beats per minute; mmHg, millimetres of mercury; TUG, timed up and go; GS, gait speed; HG, handgrip; 5-STST, 5-times sit-to-stand test.

## 4 Discussion

The aim of this study was to evaluate the relationship between a movement speed–based physical test (SARC-T) and the functional tests included in the EWGSOP2 diagnostic algorithm, which are used to estimate the probability of sarcopenia and/or its severity.

In line with COSMIN recommendations for performance-based outcome measurement instruments, we evaluated validity-related measurement properties of SARC-T using construct validity through hypothesis testing and known-groups validity. Analysis of the tests included in the diagnostic algorithm showed statistically significant differences between participants with sarcopenia and the control group in all evaluations conducted. In particular, SARC-T revealed a significant reduction in mean propulsive velocity in individuals with sarcopenia compared to matched controls, supporting its ability to discriminate between groups. A plausible interpretation is that SARC-T captures a velocity-related component of the sit-to-stand task that may be linked to neuromuscular function and power generation, which can be affected in sarcopenia. Therefore, mean propulsive velocity could reflect aspects of functional impairment that are not fully captured by completion time alone, and may provide complementary information when evaluating physical performance.

Regarding construct validity, SARC-T was significantly associated with all EWGSOP2-recommended functional tests examined, and the observed direction and magnitude of the associations were consistent with our *a priori* hypotheses. In the sarcopenia group, correlations ranged from moderate to strong across outcomes, with particularly strong associations with handgrip strength, 5-times sit-to-stand, and Timed Up and Go, while the association with gait speed test time was moderate. Similarly, in the control group, correlations ranged from moderate to strong in all cases. Overall, all *a priori* hypotheses were confirmed, supporting construct validity of SARC-T. The stronger associations observed in the sarcopenia group may indicate a wider range of functional impairment (greater variability), which can amplify correlations between related performance constructs. Alternatively, movement velocity may become more ‘limiting’ as neuromuscular reserve decreases, strengthening the coupling between SARC-T and established functional outcomes. This should be examined in larger samples and across severity strata.

The 5-STST is widely used to assess lower limb strength and predict fall risk in older adults (Zou et al., 2022). Additionally, it has been used to estimate power generated during the maneuver of standing up from a seated position. Previous studies have established minimum power threshold values required to complete the test, showing that reduced power levels are associated with frailty in the geriatric population (Alcazar et al., 2021; Baltasar-Fernandez et al., 2021). The methodology used in these studies is based on measuring the time required to complete the test, followed by applying an equation that integrates variables such as execution time, subject height, and chair height to calculate movement power. However, this approach has methodological limitations, as variability in chair height and the imprecision of manual timing with a stopwatch may compromise the reliability and comparability of results.

Although the 5-STST is adapted for the geriatric population, many older adults present comorbidities that may complicate, alter results, or even prevent the execution of physical tests included in sarcopenia diagnostic algorithms. These conditions include Parkinson’s disease (Ponsoni et al., 2023), renal failure (Duarte et al., 2024), cardiovascular diseases, type II diabetes, dementia, and respiratory diseases (Pacifico et al., 2020; Zhang et al., 2021), all of which can affect mobility and functional capacity and, consequently, the execution and accuracy of these assessments. Additionally, in individuals with chronic heart failure, the 5-STST induced a significant increase in heart rate and perceived exertion (Tanriverdi et al., 2023). In patients with pulmonary disease and cancer, this test has also been associated with oxygen desaturation and increased fatigue in individuals with sarcopenia (Tremblay Labrecque et al., 2020; Martínez-Herrera et al., 2022).

Furthermore, a strong correlation has been reported between maximum acceleration, measured via accelerometry, and performance in the 5-STST in the geriatric population (Tateoka et al., 2023). It has also been shown that the speed at which an older adult rises from a chair may be a more informative parameter for assessing muscle function than the total time required to complete a repeated sit-to-stand task (Alcazar et al., 2018b). On the other hand, the 5-STST, TUG and gait speed assessments may be subject to bias derived from human timing error, potentially limiting their reproducibility. In this context, SARC-T quantifies movement speed using a validated optoelectronic encoder and requires only two repetitions, which may reduce fatigue while providing objective and standardised measurement. These features support SARC-T as a complementary test to existing assessments of sarcopenia and physical performance and suggest potential integration into diagnostic pathways. Another relevant aspect is the difficulty of performing tests that assess sarcopenia severity, such as gait speed or TUG, in individuals with gait alterations, as may occur in people with sarcopenia and Parkinson’s disease. In such cases, freezing of gait or festinating gait could compromise test execution (José et al., 2013). Moreover, reduced handgrip strength secondary to osteoarthritis and fatigue in older adults could present a barrier to the application or interpretation of tests such as handgrip and gait speed (Vlietstra et al., 2019). From a practical standpoint, compared with time-based field tests (e.g., 5-STST, TUG, gait speed), SARC-T may offer an instrumented and standardised velocity output that could reduce manual timing error and provide complementary information on physical performance. Potential disadvantages include the need for specialised equipment and operator training, which may limit scalability in lower-resource settings.

From a feasibility and safety perspective, physiological variables (oxygen saturation, heart rate, and blood pressure) were broadly stable before and after SARC-T, and no statistically significant between-group differences were observed at baseline or immediately after testing. In the sarcopenia group, physiological responses during SARC-T were comparable to those observed during the other functional tests routinely used in sarcopenia evaluation, with no indication of a more concerning response pattern for SARC-T. These findings support the feasibility of administering SARC-T in very old adults and suggest that it can be performed without evidence of disproportionate physiological perturbation under the conditions tested (Fisher et al., 2015).

Since sarcopenia selectively affects skeletal muscle, the progressive loss of type II fibers, responsible for high-speed movements, may contribute to the reduced movement velocity observed in patients with sarcopenia (Nilwik et al., 2013; Larsson et al., 2019). This slower execution speed could imply a lower relative effort intensity during the task compared to non-sarcopenic individuals, which may partially explain the absence of marked physiological changes in this cohort. However, mechanistic interpretations should be made cautiously, and future studies incorporating objective measures of effort and exertion would be valuable.

The incorporation of an optoelectronic encoder enhances measurement accuracy by minimising assessor bias and enabling objective monitoring of performance (Ruiz-Alias et al., 2024). These features support SARC-T not only as an assessment instrument, but also as a potentially useful tool for tracking functional changes over time and for evaluating responses to interventions in clinical practice.

In clinical terms, SARC-T may be particularly valuable in situations where traditional assessments, such as the 5-STST, gait speed, or handgrip strength, are contraindicated or impractical. In older adults with advanced osteoarthritis, balance impairments, or multiple comorbidities, the low burden of a brief chair-rise velocity assessment and its feasibility in limited space may facilitate evaluation. Nevertheless, SARC-T requires a validated and calibrated measurement device, and the availability of such equipment may affect scalability in lower-resource settings. In primary care, SARC-T could be administered using a standard chair-rise task within a small clinical area, providing an objective complement when gait-based assessments are impractical. In institutional settings (e.g., long-term care), the brief two-repetition protocol may support assessment in very old adults with multimorbidity, provided that device availability and protocol standardisation are ensured.

### 4.1 Limitations

The present study has some limitations that should be considered when interpreting its results. First, participants were not stratified by age subgroups or by specific comorbidities, which may influence functional performance. The sample size was limited; larger samples would improve precision and generalisability. In addition, individual factors such as motivation, familiarity with the test, and differences in execution technique may have influenced outcomes. Another limitation of SARC-T is that it requires a validated and calibrated optoelectronic encoder, a resource that may not be available in all settings. Ensuring protocol standardisation and evaluator training is crucial to improving reproducibility. Future research should also explore the applicability of SARC-T using alternative measurement tools and evaluate additional measurement properties in broader clinical contexts.

### 4.2 Future research

In the short term, it will be a priority to complete the COSMIN evaluation of SARC-T regarding test-retest reliability and measurement error, including the standard error of measurement and the minimal detectable change, to support longitudinal interpretability. In the medium term, larger-sample studies should examine its discriminative ability using ROC curve analyses to propose operational cut-off values and estimate sensitivity and specificity in relation to EWGSOP2 classification. In the long term, it will be important to confirm its external validity across different settings and populations, conduct subgroup analyses, and assess its responsiveness in longitudinal and intervention studies, determining its usefulness for monitoring change over time.

## 5 Conclusion

In this sample of older adults, SARC-T showed moderate-to-strong associations with EWGSOP2-recommended functional tests and was able to discriminate between participants classified with sarcopenia and matched controls. In addition, under the protocol evaluated, physiological variables recorded before and after SARC-T remained broadly stable and were comparable between groups. Overall, these findings appear to support the use of SARC-T as a complementary performance-based measure for assessing physical function related to sarcopenia, particularly in settings where other tests may be less feasible.

## Data Availability

The original contributions presented in the study are included in the article/Supplementary Material, further inquiries can be directed to the corresponding author.
