# Supplementary material for: SARC-T a new physical test for sarcopenia assessment with development, validation and physiological evaluation
Source: Front Aging. 2026 Mar 2;7:1649622. doi: 10.3389/fragi.2026.1649622 (PMC12989750; doi:10.3389/fragi.2026.1649622)
Supplement: Supplementary file 1 [file Table1.docx]

**Table S1**. Physiological response to physical test.

| **Variable** | **Control group (n=38)** | | **Sarcopenia group (n=38)** | **p-value** | |  |
| --- | --- | --- | --- | --- | --- | --- |
|  | **Time Up Go** | | | | |  |
| **SpO2 baseline (%)** | 95.4 ± 1.5 | | 95.8 ± 1.3 | 0.100 | |  |
| **SpO2 final (%)** | 95.3 ± 2.0 | | 95.5 ± 1.7 | 0.289 | |  |
| **HR baseline (bpm)** | 69.6 ± 10.7 | | 67.0 ± 11.7 | 0.456 | |  |
| **HR final (bpm)** | 74.8 ± 11.0 | | 75.2 ± 10.9 | 0.425 | |  |
| **SBP baseline (mmHg)** | 134.0 ± 19.7 | | 127.6 ± 17.6 | 0.070 | |  |
| **SBP final (mmHg)** | 134.0 ± 21.0 | | 129.2 ± 20.1 | 0.156 | |  |
| **DBP baseline (mmHg)** | 73.1 ± 7.2 | | 73.7 ± 9.2 | 0.381 | |  |
| **DBP final (mmHg)** | 71.9 ± 7.3 | | 73.1 ± 9.2 | 0.269 | |  |
|  | **Gait Speed Test** | | | | |  |
| **SpO2 baseline (%)** | 95.5 ± 2.1 | | 96.1 ± 1.7 | 0.102 | |  |
| **SpO2 final (%)** | 95.4 ± 2.5 | | 96.1 ± 2.4 | 0.091 | |  |
| **HR baseline (bpm)** | 78.6 ± 12.4 | | 70.3 ± 11.3 | 0.002 | |  |
| **HR final (bpm)** | 84.6 ± 14.6 | | 77.7 ± 14.0 | 0.019 | |  |
| **SBP baseline (mmHg)** | 133.6 ± 18.8 | | 128.2 ± 16.5 | 0.094 | |  |
| **SBP final (mmHg)** | 131.9 ± 20.0 | | 125.7 ± 20.6 | 0.096 | |  |
| **DBP baseline (mmHg)** | 75.4 ± 14.3 | | 77.7 ± 23.1 | 0.303 | |  |
| **DBP final (mmHg)** | 68.6 ± 16.0 | | 70.4 ± 17.7 | 0.329 | |  |
|  | **5-STST** | | | | |  |
| **SpO2 baseline (%)** | 96.5 ± 2.1 | | 97.2 ± 1.9 | 0.120 | |  |
| **SpO2 final (%)** | 97.4 ± 3.5 | | 96.7 ± 3.9 | 0.144 | |  |
| **HR baseline (bpm)** | 73.9 ± 11.6 | | 73.2 ± 10.4 | 0.132 | |  |
| **HR final (bpm)** | 80.4 ± 11.7 | | 81.2 ± 9.2 | 0.351 | |  |
| **SBP baseline (mmHg)** | 131.5 ± 15.0 | | 128.3 ± 15.1 | 0.147 | |  |
| **SBP final (mmHg)** | 132.9 ± 16.5 | | 130.1 ± 18.7 | 0.358 | |  |
| **DBP baseline (mmHg)** | 71.3 ± 9.8 | | 70.9 ± 10.5 | 0.357 | |  |
| **DBP final (mmHg)** | 73.7 ± 7.8 | | 75.8 ± 9.0 | 0.278 | |  |
|  | | **Handgrip Test** | | | | |
| **SpO2 baseline (%)** | | 95.5 ± 3.1 | 95.1 ± 1.7 | | | 0.112 |
| **SpO2 final (%)** | | 94.4 ± 2.9 | 95.3 ± 1.4 | | | 0.264 |
| **HR baseline (bpm)** | | 74.4 ± 13.6 | 72.3 ± 10.3 | | | 0.192 |
| **HR final (bpm)** | | 80.4 ± 13.2 | 76.7 ± 13.8 | | | 0.381 |
| **SBP baseline (mmHg)** | | 130.2 ± 16.1 | 127.2 ± 12.5 | | | 0.147 |
| **SBP final (mmHg)** | | 132.5 ± 16.5 | 125.2 ± 19.6 | | | 0.420 |
| **DBP baseline (mmHg)** | | 71.9 ± 8.8 | 76.7 ± 20.1 | | | 0.436 |
| **DBP final (mmHg)** | | 72.3 ± 6.8 | 71.0 ± 16.7 | | | 0.488 |

HR: Heart Rate; SBP; systolic Blood Pressure; DBP: Diastolic Blood Pressure; 5-STST: 5-sit-to-stand-test; SpO_2_: oxygen saturation; bpm: beats per minute; mmHg: millimetres of mercury.
