# Supplementary figures and images for: SARC-T a new physical test for sarcopenia assessment with development, validation and physiological evaluation
Source: Front Aging. 2026 Mar 2;7:1649622. doi: 10.3389/fragi.2026.1649622 (PMC12989750; doi:10.3389/fragi.2026.1649622)

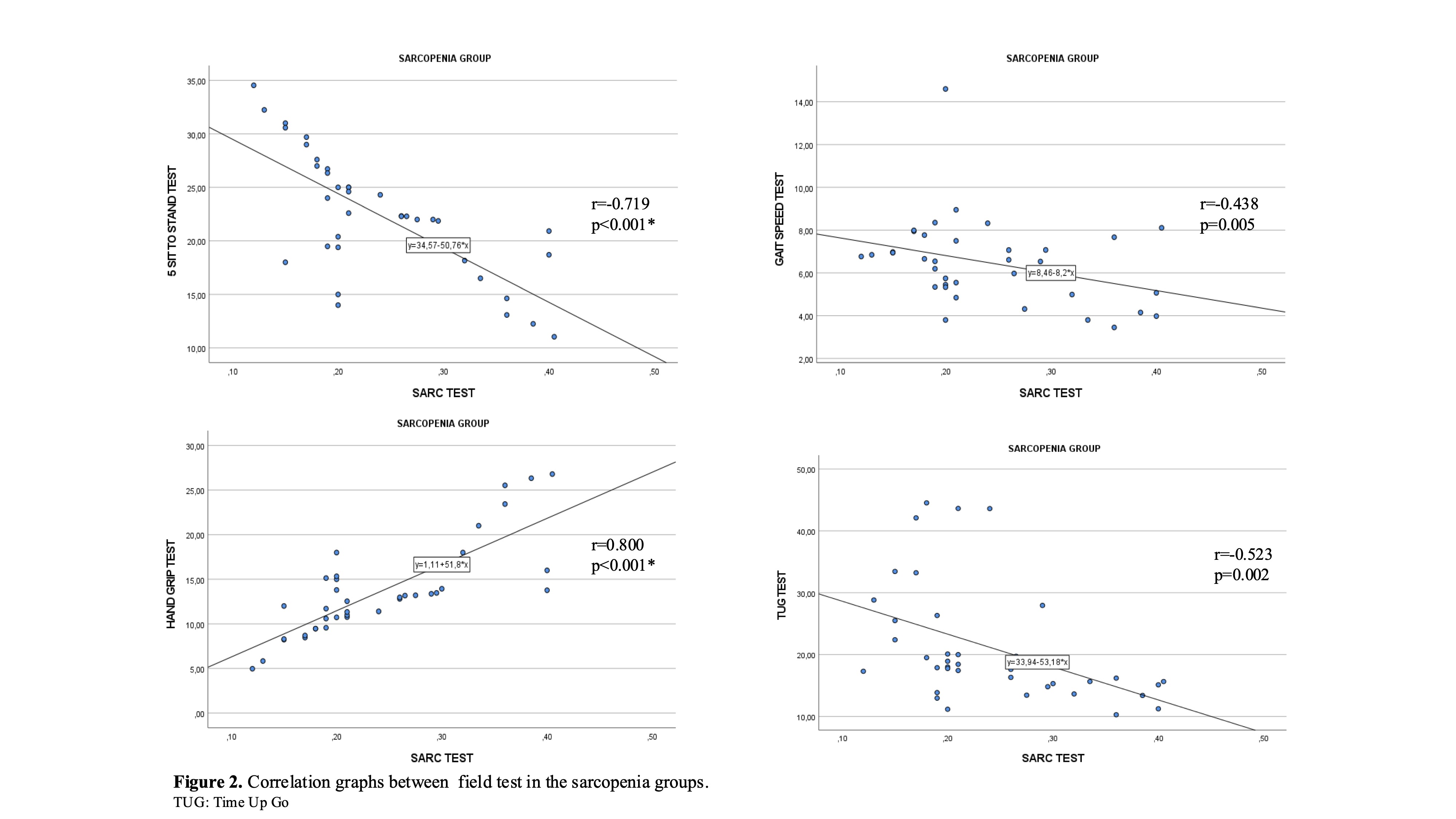

Supplement: Supplementary file 2 [file Image1.jpeg]

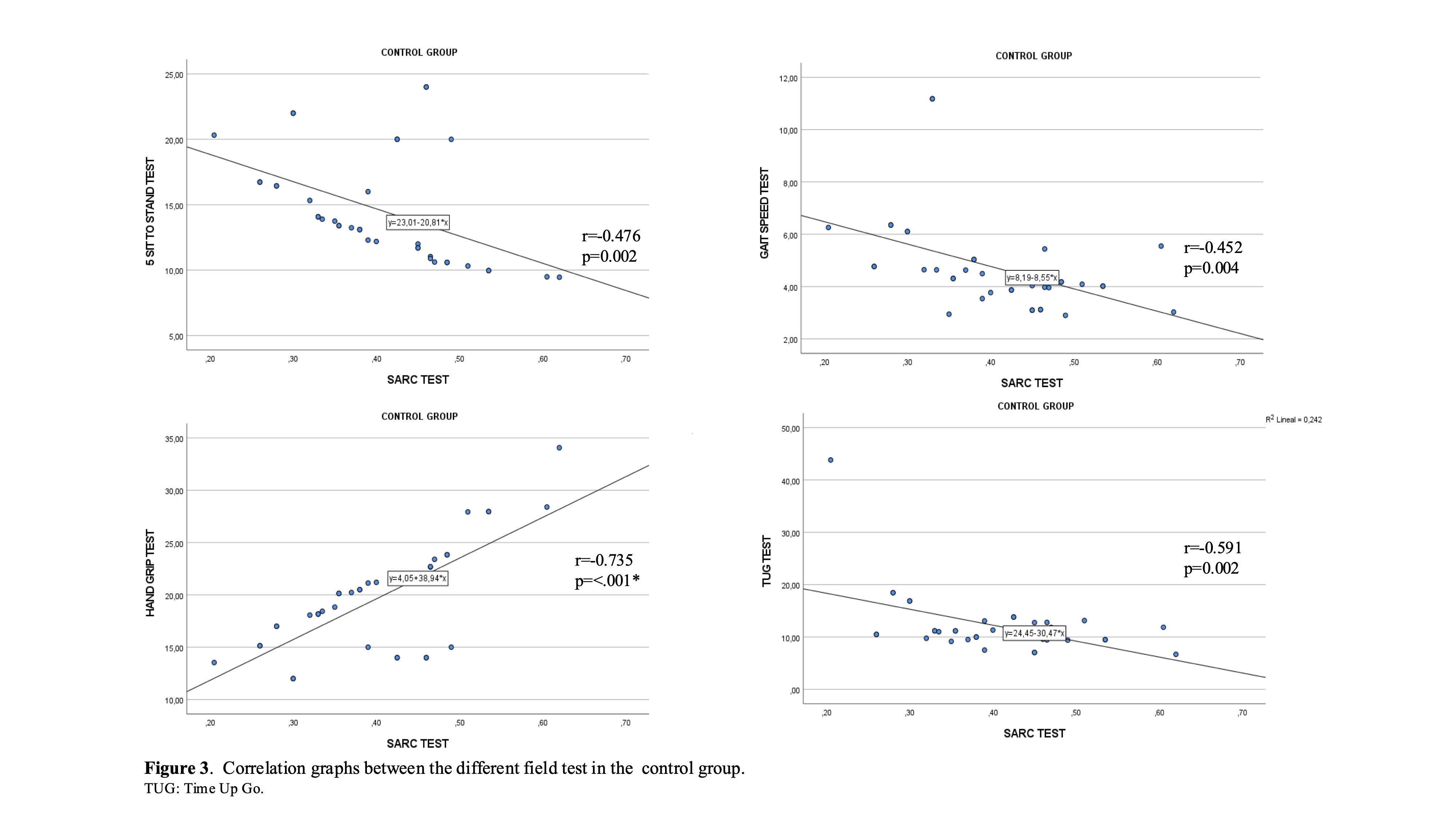

Supplement: Supplementary file 3 [file Image2.jpeg]
