# Supplementary material for: SARC-T a new physical test for sarcopenia assessment with development, validation and physiological evaluation
Source: Front Aging. 2026 Mar 2;7:1649622. doi: 10.3389/fragi.2026.1649622 (PMC12989750; doi:10.3389/fragi.2026.1649622)
Supplement: Supplementary file 4 [file Table2.docx]

**Table S2.** Physiological response across functional tests in the control group.

| **Variable** | **Control Group (n=38)** | | | | | |
| --- | --- | --- | --- | --- | --- | --- |
|  | **SARC-Test** | **TUG-Test** | **GS-Test** | **HG-Test** | **5-STST** | ***p*-value** |
| **SpO_2_ baseline (%)** | 95.5 ± 2.1 | 95.4 ± 1.5 | 95.5 ± 2.1 | 96.5 ± 2.1 | 96.1 ±1.2 | 0.115 |
| **SpO_2_ final (%)** | 95.4 ± 2.5 | 95.3 ± 2.0 | 95.4 ± 2.5 | 97.4 ± 3.5 | 96.4 ± 2.5 | 0.533 |
| **HR baseline (bpm)** | 74.4 ± 13.6 | 69.6 ± 10.7 | 78.6 ± 12.4 | 73.9 ± 11.6 | 71.9 ± 10.6 | 0.072 |
| **HR final (bpm)** | 81.4 ± 13.0 | 74.8 ± 11.0 | 84.6 ± 14.6 | 80.4 ± 11.7 | 78.4 ± 11.1 | 0.095 |
| **SBP baseline (mmHg)** | 130.5 ± 17.0 | 134.0 ± 19.7 | 133.6 ± 18.8 | 131.5 ± 15.0 | 132.5± 14.0 | 0.390 |
| **SBP final (mmHg)** | 131.5 ± 18.5 | 134.0 ± 21.0 | 131.9 ± 20.0 | 132.9 ± 16.5 | 131.9 ± 14.5 | 0.860 |
| **DBP baseline (mmHg)** | 72.8 ± 9.8 | 73.1 ± 7.2 | 75.4 ± 14.3 | 71.3 ± 9.8 | 72.3 ± 7.8 | 0.385 |
| **DBP final (mmHg)** | 73.3 ± 8.8 | 71.9 ± 7.3 | 68.6 ± 16.0 | 73.7 ± 7.8 | 71.7 ± 9.6 | 0.311 |

SpO2: oxygen saturation; HR: heart rate; SBP: systolic blood pressure; DBP: diastolic blood pressure; bpm: beats per minute; mmHg: millimetres of mercury; TUG: Timed Up and Go; GS: gait speed; HG: handgrip; 5-STST: 5-times sit-to-stand test.
